# Narrowband ultraviolet B response in cutaneous T-cell lymphoma is characterized by increased bacterial diversity and reduced *Staphylococcus aureus* and *Staphylococcus lugdunensis*

**DOI:** 10.3389/fimmu.2022.1022093

**Published:** 2022-11-11

**Authors:** Madeline J. Hooper, Gail L. Enriquez, Francesca L. Veon, Tessa M. LeWitt, Dagmar Sweeney, Stefan J. Green, Patrick C. Seed, Jaehyuk Choi, Joan Guitart, Michael B. Burns, Xiaolong A. Zhou

**Affiliations:** ^1^ Department of Dermatology, Northwestern University, Feinberg School of Medicine, Chicago, IL, United States; ^2^ Department of Biology, Loyola University Chicago, Chicago, IL, United States; ^3^ Genome Research Core, University of Illinois at Chicago, Chicago, IL, United States; ^4^ Genomics and Microbiome Core Facility, Rush University Medical Center, Chicago, IL, United States; ^5^ Division of Pediatric Infectious Diseases, Ann & Robert H. Lurie Children’s Hospital of Chicago, Chicago, IL, United States

**Keywords:** cutaneous T-cell lymphoma, microbiome, phototherapy, cancer, oncology, dermatology

## Abstract

Skin microbiota have been linked to disease activity in cutaneous T-cell lymphoma (CTCL). As the skin microbiome has been shown to change after exposure to narrowband ultraviolet B (nbUVB) phototherapy, a common treatment modality used for CTCL, we performed a longitudinal analysis of the skin microbiome in CTCL patients treated with nbUVB. 16S V4 rRNA gene amplicon sequencing for genus-level taxonomic resolution, *tuf2* amplicon next generation sequencing for staphylococcal speciation, and bioinformatics were performed on DNA extracted from skin swabs taken from lesional and non-lesional skin of 25 CTCL patients receiving nbUVB and 15 CTCL patients not receiving nbUVB from the same geographical region. Disease responsiveness to nbUVB was determined using the modified Severity Weighted Assessment Tool: 14 (56%) patients responded to nbUVB while 11 (44%) patients had progressive disease. Microbial α-diversity increased in nbUVB-responders after phototherapy. The relative abundance of *Staphylococcus*, *Corynebacterium*, *Acinetobacter*, *Streptococcus*, and *Anaerococcus* differentiated nbUVB responders and non-responders after treatment (q<0.05). Microbial signatures of nbUVB-treated patients demonstrated significant post-exposure depletion of *S. aureus* (q=0.024) and *S. lugdunensis* (q=0.004) relative abundances. Before nbUVB, responder lesional skin harboured higher levels of *S. capitis* (q=0.028) and *S. warneri* (q=0.026) than non-responder lesional skin. *S. capitis* relative abundance increased in the lesional skin of responders (q=0.05) after phototherapy; a similar upward trend was observed in non-responders (q=0.09). Post-treatment skin of responders exhibited significantly reduced *S. aureus* (q=0.008) and significantly increased *S. hominis* (q=0.006), *S. pettenkoferi* (q=0.021), and *S. warneri* (q=0.029) relative abundances compared to that of no-nbUVB patients. *Staphylococcus* species abundance was more similar between non-responders and no-nbUVB patients than between responders and no-nbUVB patients. In sum, the skin microbiome of CTCL patients who respond to nbUVB is different from that of non-responders and untreated patients, and is characterized by shifts in *S. aureus* and *S. lugdunensis*. Non-responsiveness to phototherapy may reflect more aggressive disease at baseline.

## Introduction

Cutaneous T-cell lymphoma (CTCL) is a rare non-Hodgkin’s lymphoma in which malignant T-cells infiltrate the skin. Although the etiology of CTCL remains unexplained, bacteria inhabiting the skin are understood to drive disease flares and the pathophysiology therein – specifically, *S. aureus* enterotoxin and alpha-toxin are tied to pronounced oncogenesis in CTCL (1–4). Additionally, CTCL patients have an increased risk of skin infections compared to the general population and such infections tend to be associated with disease progression (5). Influenced by local physiochemical properties and adjacent physical, autoimmune, and infectious trauma, the skin comprises a site-specific microbial niche rich with therapeutic and prognostic utility in cutaneous disease (6–8). Commensal skin bacteria play important roles in immune education and skin barrier homeostasis, and with dysbiosis comes disruption of these essential mechanisms (9). Mirroring this observation, specific changes in the skin microbiome have been associated with immune-mediated inflammatory skin conditions, including atopic dermatitis (AD) (10, 11), psoriasis (12, 13), hidradenitis suppurativa (14, 15), and vitiligo (16, 17). Nasal and gut dysbiosis have also been linked to these same diseases (15, 18). Considering recent research demonstrating CTCL patients harbor altered nasal and gut microbiota, and data revealing distinct bacterial communities distinguish healthy from lesional skin, CTCL may be a disease of global dysbiosis (19–25). While direct causality between dysbiosis and disease progression remains to be demonstrated, these findings are highly anticipated as they may precipitate innovative management strategies for CTCL patients.

Narrowband ultraviolet B (nbUVB) phototherapy is a common treatment modality for CTCL with an overall response rate of 89% in early-stage disease (26). Notably effective for skin-limited CTCL, nbUVB has been shown to shape the skin microbiome in association with disease improvement in AD and vitiligo (10, 11, 16). Interestingly, nbUVB in AD was observed to decrease lesional skin *Staphylococcus* abundance, induce clinical improvement, and enhance the anti-*S. aureus* activity and treatment effects of topical corticosteroids (11). *In vitro* studies have demonstrated ultraviolet light can suppress *S. aureus* superantigen production (27). Furthermore, excimer laser-mediated increases of *Cyanobacterium* in AD skin may reflect improved skin water content (10), thereby supporting the hypothesis that non-*Staphylococcus* taxa also play an important role in the homeostasis of skin health and disease.

We conducted a longitudinal study of the bacterial communities populating the lesional and non-lesional skin of CTCL patients treated with nbUVB phototherapy. This early effort to detail a therapeutic-microbial relationship in CTCL achieves two aims: first, to assess the impact of nbUVB on the CTCL skin microbiome; and second, to provide a comprehensive framework for evaluating the biological relevance of the microbial differences separating healthy and diseased skin.

## Materials and methods

### Participants

Ethical approval was obtained from the Northwestern University Institutional Review Board (STU00209226). In compliance with the Declaration of Helsinki, 40 patients with clinically- and biopsy-confirmed CTCL, as reviewed by an expert dermatopathologist (JG), were consented and enrolled in the study between 2019 and 2021. Specimen and data collection were performed at the Northwestern University Cutaneous Lymphoma specialty clinic. Clinical staging and modified Severity Weighted Assessment Tool (mSWAT) were assessed by the principal investigator (XAZ) at sample collection.

**Figure 1:**
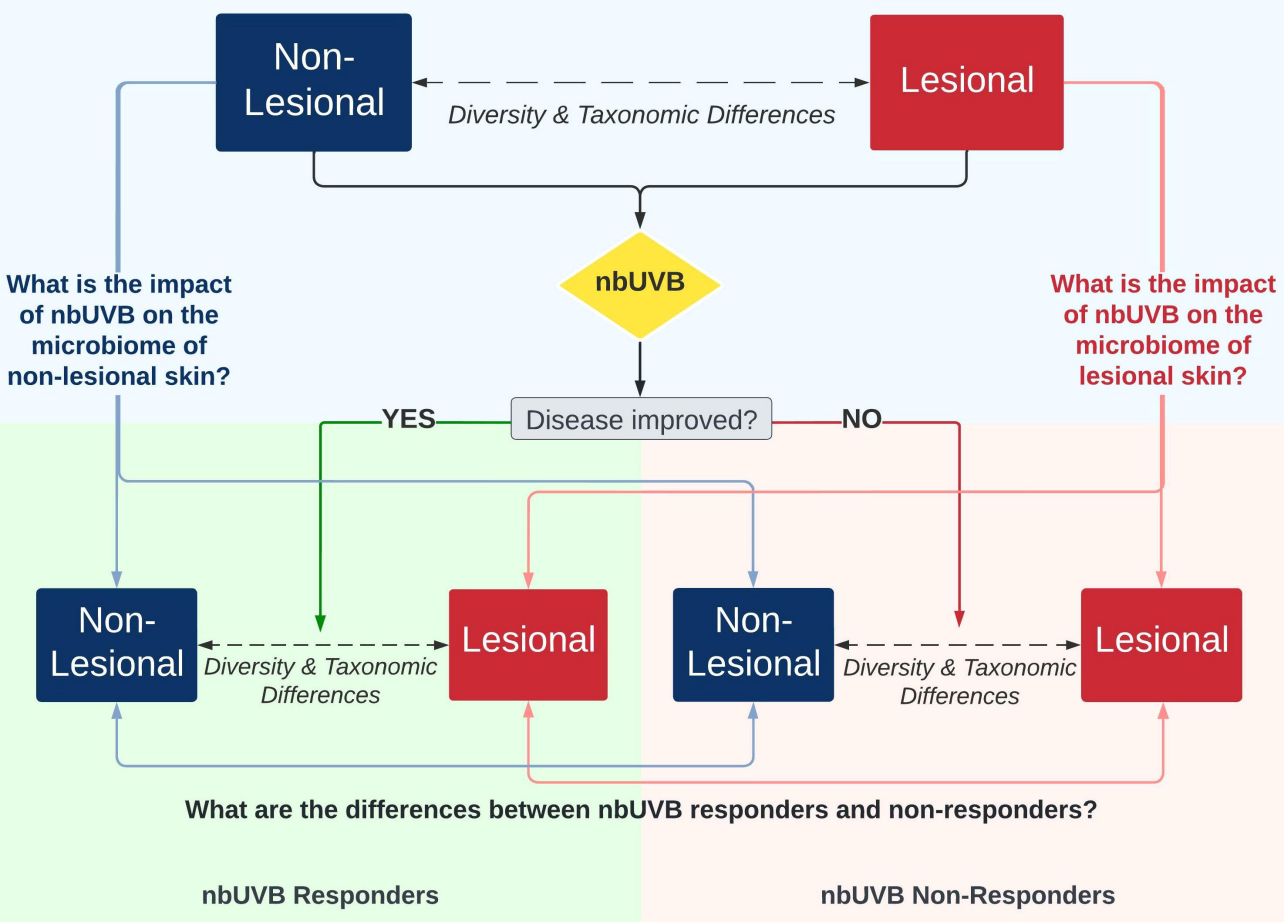
Investigational schemata for evaluating the skin microbiome of lesional and non-lesional cutaneous T-cell lymphoma (CTCL) skin in response to narrowband ultraviolet B (nbUVB) phototherapy. Differences in microbial community diversity and taxonomic abundance between responders and non-responders are illustrated in **Figures 2**–**5**.

**Figure 1** illustrates the study design. Twenty-five patients were prescribed a treatment regimen involving nbUVB phototherapy; the remaining 15 participants used non-nbUVB standard-of-care treatments. In total, 28 patients used topical treatments, of which 89% (n=25) were using topical corticosteroid monotherapy, 14 patients used systemic treatments, and 8 were treatment naïve. The great majority of patients (85%) had been stable on these therapies for minimum 24 months prior to study enrollment. Patients who used antibiotics in the 4 weeks prior to sample collection were excluded.

### 16S rRNA amplification and sequencing

Genomic DNA was prepared for sequencing using a two-stage amplicon sequencing workflow, as described previously (28). Genomic DNA was PCR-amplified using primers targeting the V4 region of microbial 16S rRNA genes. The primers, 515F modified and 806R modified, contained 5’ linker sequences compatible with Access Array primers for Illumina sequencers (Fluidigm; South San Francisco, CA) (29). PCRs were performed in a total volume of 10μL using MyTaq™ HS 2X Mix (Bioline; Memphis, TN), primers at 500nM concentration, and approximately 1,000 copies per reaction of a synthetic double-stranded DNA template (described below). Thermocycling conditions were 95°C for 5’ (initial denaturation), followed by 28 cycles of 95°C for 30 sec, 55°C for 45 sec, and 72°C for 30 sec. The second-stage PCR reaction contained 1μL of PCR product from each reaction and a unique primer pair of Access Array primers. Thermocycling conditions were 95°C for 5’ (initial denaturation), followed by 8 cycles of 95°C for 30 sec, 60°C for 30 sec, and 72°C for 30 sec. Libraries were pooled and sequenced on an Illumina MiniSeq sequencer (Illumina; San Diego, CA) with 15% phiX spike-in and paired-end 2x153 base sequencing reads.

A synthetic double-stranded DNA spike-in was created as a gBLOCK by Integrated DNA Technologies (IDT; Coralville, IA). The design basis was a 999 base pair (bp) region of the 16S rRNA gene of *Rhodanobacter denitrificans* strain 2APBS1^T^ (NC_020541) (30). Portions of V1, V2, and V4 variable regions were replaced by eukaryotic mRNA sequences (*Apostichopus japonicus* glyceraldehyde-3-phosphate dehydrogenase mRNA, HQ292612 and *Strongylocentrotus intermedius* glyceraldehyde-3-phosphate dehydrogenase mRNA, KC775387). Primer sites were preserved, and the overall length of the synthetic DNA did not differ from the equivalent *R. denitrificans* fragment. PCR amplicons generated from this synthetic DNA do not differ in size from bacterial amplicons and can only be identified and removed through post-sequencing bioinformatics analysis. The sequence can be accessed *via* GenBank (OK324963).

### Basic processing

Sequencing resulted in a total of 23,825,736 reads with an average of 42,698 reads per sample. Forward (F) and reverse (R) reads were trimmed using cutadapt v3.5 to remove primer sequences (31). All reads were trimmed and filtered based on quality using the default parameters within dada2 v1.22 (32) with the following modification: the truncLen parameter was disabled, and maxEE for all reads was set to 1,1. The error models were initiated using 5x10^8^ randomized bases for the F and R sets. Following subsequent denoising with dada2’s divisive partitioning machine learning approach, F and R amplicon sequence variant (ASV) read pairs were merged with a maxMismatch of 0 and a minOverlap of 8 bases. Merged pairs that did not fall within the expected length window of 252-254 bases were removed. Chimeric sequences were identified and removed using a *de novo* approach within dada2 assessing the entire sequence pool as a whole. ASVs were assigned taxonomy using DECIPHER v2.22 with the SILVA v138 training set (33, 34). The synthetic spike-in sequences were then removed from the dataset. The decontam R package v1.14 was used to identify potentially contaminating sequences, using environmental control samples collected at experimental sample collection (35). Suspected contaminant ASVs above the default prevalence threshold of 0.1 were classified as potential environmental artifacts and removed from the dataset. An abundance filter of 0.1% and a prevalence filter of 10% were applied. Following QC processing, decontamination, and filtering, there were a total of 5,862,546 merged read pairs with an average of 17,042 per sample. To maximize data retention, while removing uninformative patient samples, only samples with minimum 1000 reads following processing were retained. Patient samples were paired across disease status (lesional, non-lesional) and time (pre-nbUVB, post-nbUVB). Only complete patient-matched sets were retained for analysis.

### *Tuf2* amplicon next generation sequencing

Genomic DNA was PCR amplified with primers CS1_tuf2-F (ACACTGACGACATGGTTCTACAACAGGCCGTGTTGAACGTG) and CS2_tuf2-R (TACGGTAGCAGAGACTTGGTCTACAGTACGTCCACCTTCACG) (36, 37) targeting the *Staphylococcus tuf* gene. Amplicons were generated using a two-stage PCR amplification protocol, as previously described (28). First stage PCR amplifications were performed in 10μL reactions in 96-well plates, using MyTaq HS 2X mastermix (Bioline). PCR conditions were 95°C for 5 min, followed by 28 cycles of 95°C for 30 sec, 55°C for 30 sec and 72°C for 60 sec. Second-stage reactions using Access Array primers were performed as described above. Samples were pooled, purified, and sequenced on an Illumina MiSeq with 10% phiX spike-in and paired-end 2x300 base sequencing reads (*i.e.*, V3 chemistry). Library preparation, pooling, and sequencing were performed at the Genome Research Core within the Research Resources Center at the University of Illinois at Chicago.

### *Tuf2* amplicon processing

*Tuf2* sequencing generated a total of 27,022,394 reads with an average of 57,372 raw reads per sample. Primer trimming and denoising were accomplished using the same procedure as above for the 16S amplicon data with the following modifications: during filtering and trimming, the maxEE for all reads was set to 3,3 due to their increased length, read merging used default parameters, and the read length cutoff window range was 400-500 nucleotides for merged read pairs. Following processing there were a total of 4,496,566 reads retained for an average sample read count of 9,547. ASVs were taxonomically annotated using BLASTn alignments against NCBI prokaryotic (nr) refseq database (online access 14 June 2022). Only taxa that were within the *Staphylococcus* genus were retained.

### Statistical analyses

The samples in the cleaned ASV table were visually evaluated using phyloseq v1.38 (38). α-diversity metrics were generated using the ASV table rarefied to 1000 sequences. Differences in α-diversity (Observed OTUs, Chao1, Shannon, and Simpson indices) between patient sets were calculated using Wilcoxon rank-sum non-paired tests from the stats R package (39) while differences within patient-matched samples were calculated using Pairwise Wilcoxon rank-sum tests. β-diversity metrics were generated using the rarefied ASV table. Principal coordinate analysis (PCoA) with Bray-Curtis dissimilarity was performed to identify β-diversity using an ADONIS2 method (permutations = 500) of vegan v2.5.7 (40). To verify the PCoA findings, “createDataPartition” function from the Caret package v6.0.93 was used to split the data into training and test sets (41) and a supervised machine-learning algorithm Random Forest from the random Forest package v 4.7.1.1 was applied for a 10-fold cross-validation in R. The model was created using 1500 trees (42). To evaluate the model, receiver operating characteristic curves and area under the curve (AUC) values were generated using the “roc” function of the R pROC package v1.18.0 (43). Differential abundance analysis was conducted by metagenomeSeq v1.36 (44) using the non-rarefied ASV table, with ASVs removed if they had less than 4 counts or a prevalence below 10% across the sample set. A zero-inflated Gaussian (ZIG) log-normal model was implemented using the “fitFeatureModel” function of the metagenomeSeq R package to compare abundance of taxa among different groups. To further refine the differential taxa results and to reduce the likelihood of false positives, a second method, edgeR v3.36 (45), was applied to the same ASV table. For both the metagenomeSeq and edgeR results, significant ASVs were only considered if they achieved a false discovery rate (FDR)-adjusted p-value of <0.05 (q-value) by both methods.

The post-process *tuf2* ASV and taxa table was filtered using phyloseq v1.38 (38). To identify the most abundant *Staphylococcus* species, filters of minimum 20% prevalence and 4 ASVs were applied. After centered log-ratio (CLR) transformation, the final ASV table including only the *Staphylococcus* species present at 20% abundance or greater was used for statistical analysis. Differences in abundance between patient groups were calculated using Wilcoxon rank-sum non-paired tests from the stats R package (39) while Pairwise Wilcoxon rank-sum tests were used to calculate differences between patient-matched samples. Differential abundance analysis was conducted using the CLR transformed abundance table. A ZIG log-normal model as described above was implemented to compare abundance of the top 8 *Staphylococcus* species among different groups. Significant ASVs were only considered if they achieved a FDR of <0.05.

## Results

### Patient characteristics

**Table 1** summarizes patient characteristics. Of the 25 CTCL patients treated with nbUVB, 20 were diagnosed with mycosis fungoides, 2 with Sézary syndrome, and 3 with other CTCL subtypes. The patients were predominately male (n=17, 68.0%) and Caucasian (n=19, 76.0%). Fifteen sex-, age-, and race-matched patients who were not treated with nbUVB phototherapy (NT) were also included (**Table 2**). On average, nbUVB-treated participants were evaluated after 6.2 months (range 1.6-14.7) of treatment, at which time 14 patients (56.0%) had improved disease (Responders [R]; mean mSWAT change -18.5) and 11 (44.0%) had progressive disease (Non-Responders [NR]; mean mSWAT change +11.4). There were no significant differences in demographic or clinical characteristics between R and NR (p>0.05).

**Table 1:** Patient demographic and clinical characteristics.

	Responders	Non-responders	*p-value*
**N**	14	11	
**Mean age (range), years** ^†^	58 (35-78)	57 (36-72)	0.857
**Sex (%)** ^‡^			1.000
* Male*	10 (71.4)	7 (63.6)	
* Female*	4 (28.6)	4 (36.4)	
**Race (%)** ^‡^			0.333
* White*	11 (78.6)	8 (72.7)	
* African American*	1 (7.1)	3 (27.3)	
* Other*	2 (14.3)	0 (0.0)	
**FST (%)** ^‡^			0.623
* Light (I-III)*	12 (85.7)	8 (72.7)	
* Dark (IV-VI)*	2 (14.3)	3 (27.3)	
**CTCL Subtype (%)** ^‡^			0.774
* Mycosis fungoides*	12 (85.7)	8 (72.7)	
* Sézary syndrome*	1 (7.1)	1 (9.1)	
* Other CTCL*	1 (7.1)	2 (18.2)	
**Stage (%)** ^‡^			0.115
* Early (IA-IIA)*	11 (78.6)	5 (45.5)	
* Late (IIB-IVB)*	3 (21.4)	6 (54.5)	
**Non-nbUVB treatments (%)** ^‡^			0.927
* Skin-directed only*	7 (50.0)	4 (36.4)	
* Systemic only*	1 (7.1)	1 (0.1)	
* Skin-directed and systemic*	3 (21.4)	4 (36.5)	
* None*	3 (21.4)	2 (18.2)	
**Mean change in mSWAT,** **pre- versus post-nbUVB (range)**	-18.5 (-52 - -1)	11.4 (2-79)	0.002^†^

CTCL, cutaneous T-cell lymphoma; FST, Fitzpatrick skin phototype; mSWAT, modified Severity Weighted Assessment Tool; nbUVB, narrowband ultraviolet B.

^†^Independent T-test, **^‡^** Fisher exact test.

**Table 2:** Demographic and clinical characteristics of patients not treated with nbUVB (NT) compared to nbUVB responders (R) and non-responders (NR).

	No nbUVB (NT)	R vs NT, *p-value*	NR vs NT, *p-value*
**N**	15		
**Mean age (range), years^†^**	64 (37-83)	0.307	0.188
**Sex (%)^‡^**		1.000	1.000
* Male*	10 (66.7)		
* Female*	5 (33.3)		
**Race (%)^‡^**		0.791	0.279
* White*	13 (86.7)		
* African American*	0 (0.0)		
* Other*	2 (13.3)		
**FST (%)^‡^**		0.598	0.279
* Light (I-III)*	14 (93.3)		
* Dark (IV-VI)*	1 (6.7)		
**CTCL Subtype (%)^‡^**		1.000	1.000
* Mycosis fungoides*	11 (73.4)		
* Sézary syndrome*	2 (13.3)		
* Other CTCL*	2 (13.3)		
**Stage (%)^‡^**		0.009	0.419
* Early (IA-IIA)*	4 (26.6)		
* Late (IIB-IVB)*	11 (73.4)		
**Treatments (%)^‡^**		0.884	0.925
* Skin-directed only*	7 (46.7)		
* Systemic only*	0 (0.0)		
* Skin-directed and systemic*	5 (33.3)		
* None*	3 (20.0)		
**Mean change in mSWAT,** **pre- versus post-nbUVB (range)^†^**	3.2 (-10 - 37)	0.807

CTCL, cutaneous T-cell lymphoma; FST, Fitzpatrick skin phototype; mSWAT, modified Severity Weighted Assessment Tool; nbUVB,narrowband ultraviolet B.

^†^Independent T-test, **^‡^** Fisher exact test.

Treated patients were prescribed nbUVB two or three times per week. All patients endorsed using standard-of-care topical (n=31) and systemic (n=14) CTCL treatments (**Supplemental Table S1**). Hypercholesterolemia (n=21) and hypertension (n=17) were the most common comorbidities across the entire cohort. There were no significant differences in treatment profiles or comorbidity prevalence amongst R, NR, and NT groups (p=0.822 and p=0.656, respectively).

### Biodiversity and community richness modulated by nbUVB

**Figure 2:**
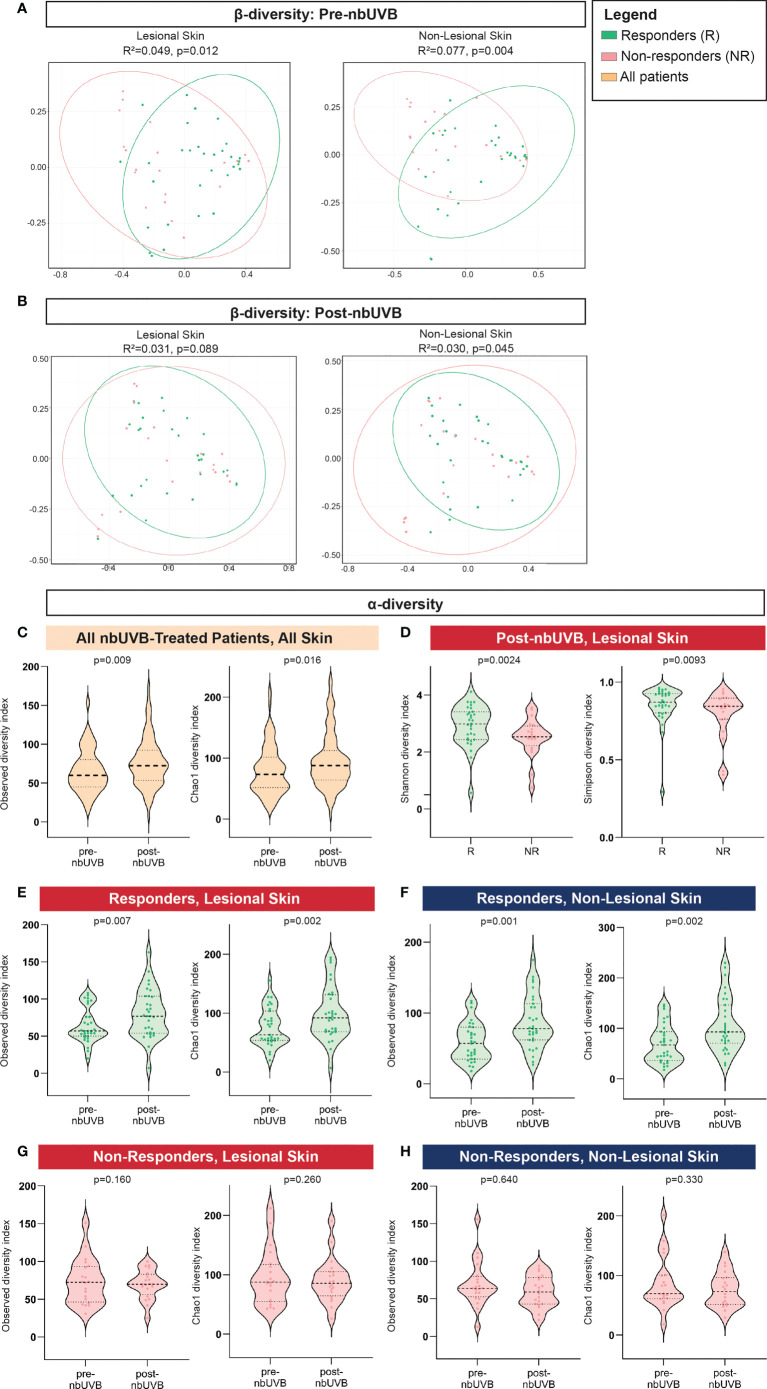
Distinct microbial communities comprise the skin microbiota of nbUVB responders and non-responders before and after nbUVB. **(A)** Bray-Curtis dissimilarity indices demonstrated Responder **(R)** and Non-Responder (NR) bacterial skin communities were distinct prior to nbUVB in lesional and non-lesional skin. **(B)** R versus NR post-nbUVB community differences approached significance in lesional skin, but reached significance in non-lesional skin. **(C)** Amongst all nbUVB-treated patients, α-diversity increased after phototherapy. **(D)** Following nbUVB, α-diversity of lesional skin was higher in R than NR. **(E)** α-diversity of R lesional skin and **(F)** non-lesional skin significantly increased after phototherapy. **(G)** No pre- versus post-nbUVB α-diversity differences were noted in NR lesional or **(H)** non-lesional skin. Thick dashed horizontal black lines indicate group median; thin dashed lines indicate 1^st^ and 3^rd^ quartiles.

The pre-nbUVB microbial communities of R and NR were distinct in both lesional (Bray-Curtis index; R^2^ = 0.049, p=0.012; AUC=0.852) and non-lesional skin (R^2^ = 0.077, p=0.004; AUC=0.796) (**Figure 2A**). After phototherapy, R and NR non-lesional microbiota remained distinct (R^2^ = 0.030, p=0.045; AUC=0.852), but lesional communities became non-distinct (R^2^ = 0.031, p=0.089; AUC=0.759) (**Figure 2B**). As expected, the PCoA plots show between-group overlap because the samples are all from the same microbial niche.

Phylogenetic diversity increased in all treated patients’ skin after phototherapy (observed p=0.009; Chao1 p=0.016) (**Figure 2C**). Post-nbUVB, lesional α-diversity was significantly greater in R than in NR (Shannon p=0.0024; Simpson p=0.0093) (**Figure 2D**). This difference corresponded to a significant increase in α-diversity after nbUVB in R skin (lesional: observed p=0.007, Chao1 p=0.002; non-lesional: observed p=0.001, Chao1 p=0.002) (**Figures 2E, F**) while no significant changes were noted in NR skin (lesional: observed p=0.160, Chao1 p=0.260; non-lesional: observed p=0.640, Chao1 p=0.330) (**Figures 2G, H**).

### Decreased *Staphylococcus*, increased *Acinetobacter* differentiate nbUVB responsiveness after phototherapy

**Figure 3:**
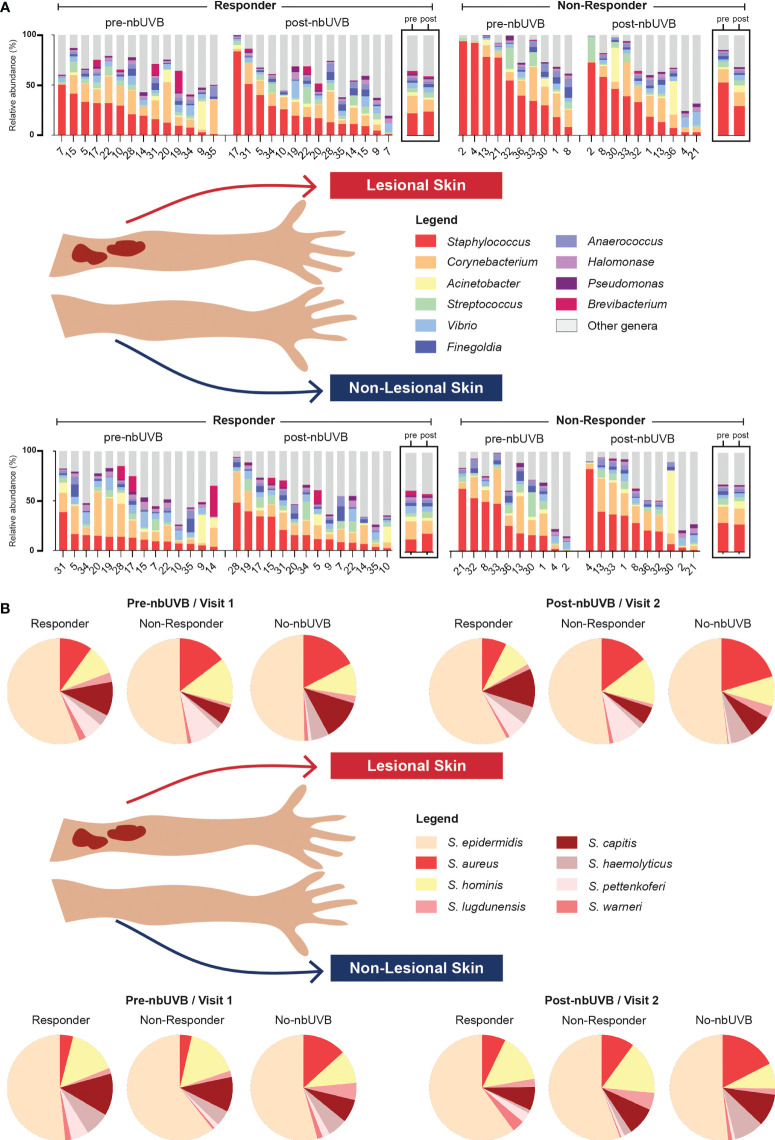
Microbial communities of CTCL lesional and non-lesional skin are predominated by different taxa. **(A)** At the taxonomic level of genus, *Staphylococcus*, *Corynebacterium*, and *Acinetobacter* were the most prevalent and abundant genera present in all treated samples. Bar charts indicate relative abundance (%) of the 10 most abundant genera populating the skin of each patient; boxed graphs reflect group mean relative abundance at pre-nbUVB (left) and post-nbUVB (right). Subject IDs are indicated along the x-axis and ordered to best visualize the distribution of relative abundances in descending order. Of note, this figure is not intended to illustrate statistically significant differences. **(B)** Staphylococcal speciation across all samples revealed a majority presence of *S. epidermidis*, *S. aureus*, and *S. hominis*. Pie charts reflect mean relative abundance of each species identified across R, NR, and no-nbUVB patient groups.

**Figure 4:**
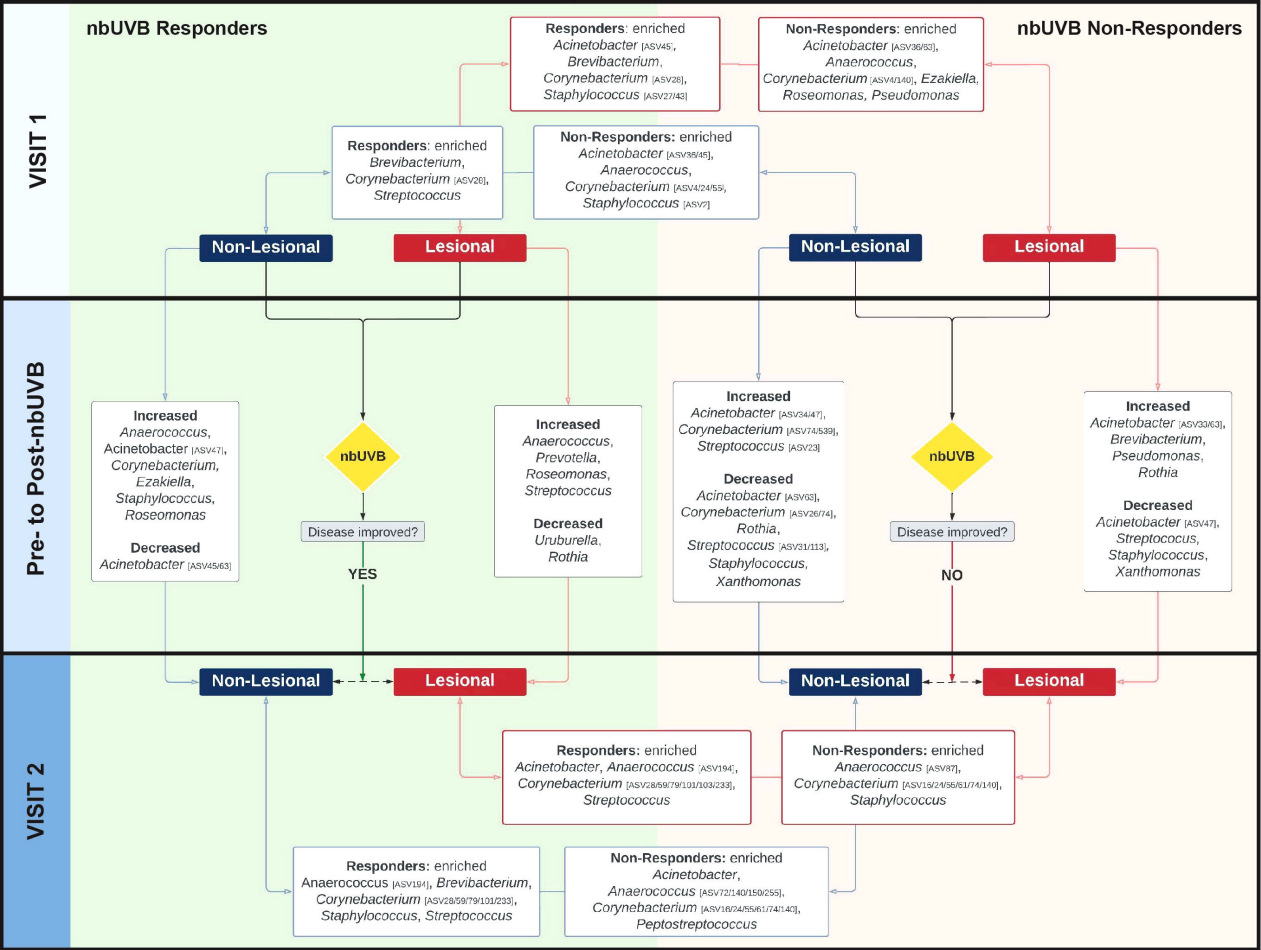
The skin microbiome changes after exposure to nbUVB phototherapy, as demonstrated by shifts at the taxonomic level of genus. Map of genus-level taxa abundances differentiating responders **(R)**and non-responders (NR) at lesional and non-lesional sites. Panels represent specific analyses: pre-nbUVB (top panel), pre- versus post-nbUVB (middle panel), and post-nbUVB (bottom panel). In cases of mixed responses at the genus level, specific ASVs identified on differential analysis are noted. Bidirectional arrows indicate the skin sites being compared at the same time point (e.g., R/lesional skin/pre-nbUVB versus NR/lesional skin/pre-nbUVB); unidirectional arrows indicate longitudinal shifts (e.g., R/lesional skin/pre-nbUVB versus R/lesional skin/post-nbUVB).

The genera *Staphylococcus*, *Corynebacterium*, and *Acinetobacter* predominated all analyzed specimens (**Figure 3A**). Differential analyses revealed significant genus-level shifts that distinguished R and NR, and lesional and non-lesional skin in response to phototherapy (q<0.05). In addition to genus-level taxonomic shifts, several specific ASVs within these genera provided opposing abundance shifts across the patient sample set. **Figure 4** summarizes these genus-level taxon-by-taxon differences with specific reference to the unique ASV shifts, as appropriate.

#### Pre-nbUVB treatment

Before phototherapy, *Brevibacterium*, *Staphylococcus* ASV27, and *Staphylococcus* ASV43 were significantly more abundant in eventual R relative to eventual NR across both lesional and non-lesional sites. Comparing non-lesional samples, *Streptococcus* was more abundant in R and *Staphylococcus* ASV2 was more abundant in NR. Mixed responses among several *Corynebacterium* ASVs were observed: ASV28 was more abundant in R, while ASV4 was more abundant in NR. At lesional sites, *Acinetobacter* ASV45 was greater in R than NR while *Acinetobacter* ASV36 was depleted in R; at non-lesional sites, both *Acinetobacter* ASV45 and ASV36 were more abundant in R.

#### Comparing pre- and post-nbUVB treatment

Pre- versus post-phototherapy comparisons of R lesional skin showed increased abundance of *Streptococcus*, *Anaerococcus*, and *Prevotella* after nbUVB treatment (**Figure 4**). In contrast, NR lesional skin demonstrated a reduction of *Streptococcus*, *Staphylococcus*, and *Acinetobacter*. R and NR shared some non-lesional genus-level similarities: mixed changes were observed amongst *Acinetobacter* ASVs (decreased ASV63; increased ASV47) and several *Corynebacterium* ASVs were more abundant (ASV16/233 in R, ASV74/539 in NR). While R patient samples revealed increased relative abundance of lesional *Streptococcus* ASV31, NR lesional skin exhibited a loss of this ASV.

#### Post-nbUVB treatment

Finally, post-nbUVB R lesional skin was differentiated from NR lesional skin by greater relative abundance of *Acinetobacter* and *Streptococcus*, and lower relative abundance of *Staphylococcus*. A mixed abundance distribution across several *Corynebacterium* ASVs was observed in R compared to NR skin: increased ASV101/103/233/28/59/79, and decreased ASV140/16/24/55/61/74. The pre-nbUVB differences in *Brevibacterium* and *Corynebacterium* identified between R and NR non-lesional skin persisted after nbUVB.

With ASVs collapsed by genus, taxon-by-taxon analysis revealed *Streptococcus* comprised a larger relative abundance in R than NR lesional skin (p=0.008, q=0.08) after phototherapy; this comparison approached significance in non-lesional skin (p=0.047, q=0.260). In the pre- versus post-nbUVB analysis, loss of *Corynebacterium* across all lesional sites trended towards significance (p=0.033, q=0.330).

### Genus-level differences distinguishing nbUVB-treated patients from no-nbUVB patients

No pre- versus post-nbUVB changes in NT α- or β-diversity were observed (Shannon p=0.51; Bray-Curtis R^2^ = 0.007, p=0.425). Differential analysis of microbial profiles at the genus level between treated and NT patients revealed phototherapy has similar effects on the skin microbiota regardless of disease response. Comparison of R and NT at visit 2 demonstrated *Acinetobacter* and *Corynebacterium* (ASV24/55) were more abundant in NT, whereas *Corynebacterium* ASV233 and *Streptococcus* were more abundant in R. Similarly, NR versus NT analysis indicated *Acinetobacter* and *Corynebacterium* (ASV16) were more abundant in NT. *Peptostreptococcus* and *Anaerococcus* were more abundant in NR.

### Shifts in *Staphylococcus* species abundance are associated with nbUVB responsiveness

Given the changes in relative abundance of *Staphylococcus* at the genus level and considering the importance of these species to skin health, we next assessed *Staphylococcus* species-level relative abundance within our samples using tuf amplicon sequencing. The most prevalent and abundant species were *S. epidermidis*, *S. aureus*, and *S. hominis* (**Figure 3B**).

**Figure 5:**
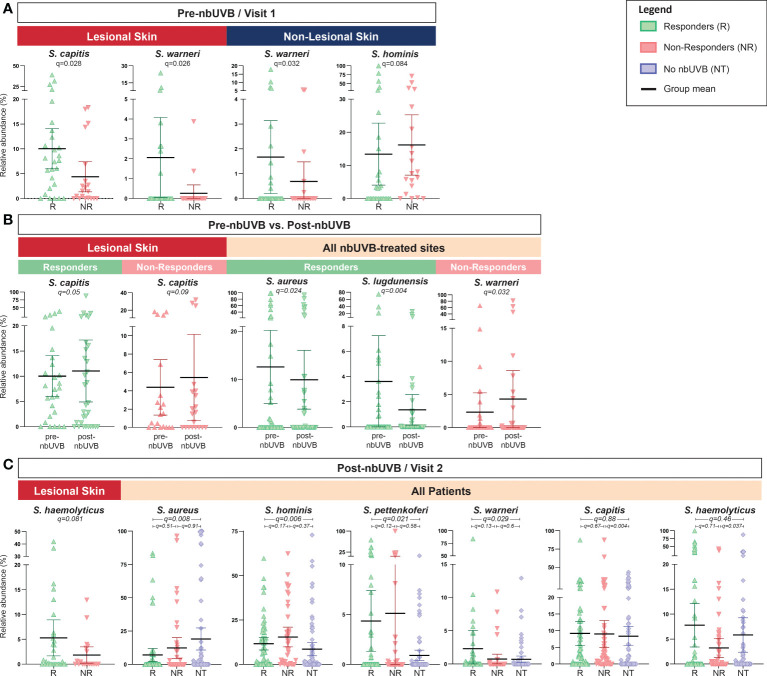
*Staphylococcus* species abundance differentiates nbUVB responders, nbUVB non-responders, and no-nbUVB patients. Differential analysis demonstrated significant shifts in various staphylococcal species including *S. aureus*, *S. lugdunensis*, *S. hominis*, and *S. haemolyticus* characterize R, NR, and no-nbUVB (not treated; NT) skin **(A)** pre-nbUVB, **(B)** pre- versus post-nbUVB, and **(C)** post-nbUVB. Black horizontal bars indicate group means with 95% confidence interval.

Analysis of pre-nbUVB R versus NR revealed greater relative abundances of *S. capitis* (q=0.028) and *S. warneri* (q=0.026) characterized R lesional skin (**Figure 5A**). Pre-treatment non-lesional skin also exhibited greater relative abundance of *S. warneri* in R than NR (q=0.032), while *S. hominis* relative abundance trended higher in NR than R (q=0.084) (**Figure 5A**). Pre- versus post-nbUVB assessment demonstrated *S. capitis* communities were significantly more abundant in R lesional skin (q=0.05) and trended higher in NR lesional skin (q=0.09) after phototherapy (**Figure 5B**). Across all sites, relative abundance of *S. aureus* (q=0.024) and *S. lugdunensis* (q=0.004) significantly decreased after phototherapy in R while *S. warneri* increased in NR (q=0.032). Lastly, post-nbUVB *S. haemolyticus* relative abundance trended higher in R compared to NR lesional skin (q=0.081) (**Figure 5C**). Post-treatment R versus NT analysis revealed R skin was characterized by significantly less abundant *S. aureus* (q=0.008) and significantly more abundant *S. hominis* (q=0.006), *S. pettenkoferi* (q=0.021), and *S. warneri* (q=0.029). NR versus NT comparisons revealed *S. capitis* relative abundance was greater in NR (q=0.004) while *S. haemolyticus* was more abundant in NT (q=0.037).

## Discussion

The antigenic stimulation provided by skin microbiota and bacterial toxins is likely one of several phenomena that, in concert with chronic skin inflammation, drives CTCL progression. Our observations suggest that successful treatment with phototherapy is associated with a microbial signature unique from that of untreated disease and phototherapy-refractory disease. This finding could suggest skin microbiota may play a role in nbUVB-responsiveness; conversely, improved disease may be characterized by a distinct microbial signature outside of any nbUVB-related effects. The increased α-diversity of nbUVB responders’ skin also supports the hypothesis that phototherapy helps recoup microbial richness that may be lost in CTCL. This change could be explained by targeted attenuation of abundant taxa, such as *S. aureus* and *S. lugdunensis*, as seen in this dataset. Because the influence of cutaneous microbes on skin health is manifold, these findings underscore the potential role of commensal skin bacteria in CTCL pathogenesis and the therapeutic applications therein.

The current literature strongly supports a key role for *S. aureus* in CTCL progression (2, 3, 46); however, little is known about the influence of other staphylococcal species. We noted baseline genus-level *Staphylococcus* and species-level *S. capitis* and *S. warneri* abundances were higher in patients that eventually responded to phototherapy. These findings suggest commensal staphylococci abundance could help prognosticate disease responsiveness to phototherapy. Patient heterogeneity may limit the applicability of this theory, which warrants further study. Moreover, fewer significant differences were found between the species-level *Staphylococcus* profiles of non-responders and no-nbUVB patients than responders and no-nbUVB patients. This observation could indicate that the benefit of phototherapy in CTCL may be derived from the modulation of non-*S. aureus* species. Similarly, *S. epidermidis* trended higher in R after phototherapy across lesional and non-lesional skin. While the importance of coagulase-negative staphylococci to CTCL disease activity has yet to be determined, quorum sensing – as has been discussed in AD – could explain this finding (47). Further, very recent research revealed *S. warneri* exhibits methicillin-resistant *S. aureus* quorum sensing activity that helps protect skin from atopic and necrotic damage (48). Lastly, *S. epidermidis* – a very common commensal coagulase-negative staphylococcal species – contributes to skin microbial homeostasis through its ability to inhibit *S. aureus* growth *via* the production of antimicrobial peptides (49) and interfering with *S. aureus* biofilm production (50). Additionally, *S. epidermidis* has been shown to have anti-tumor properties in UV-induced skin cancers (51) and can suppress cutaneous inflammation by activating regulatory T-cells (52).

Other notable genera from this analysis included *Acinetobacter*, *Corynebacterium*, *Brevibacterium*, and *Streptococcus.* As *in vitro* studies have demonstrated *Acinetobacter* can induce anti-inflammatory IL-10 expression in both monocytes and keratinocytes (53), our observation that *Acinetobacter* abundance was greater in responder than non-responder lesional skin after phototherapy suggests direct linkages exist between skin microbiota and the local inflammatory microenvironment in CTCL. Moreover, little is known about the effect of nbUVB on skin microbiota. Increased abundance of healthy skin commensal flora (e.g., *Anaerococcus*, *Streptococcus*, and *Acinetobacter*) in lesional skin after phototherapy may reflect the reconstitution of a balanced skin microbiome. Increased *Streptococcus* abundance has been associated with successful treatment of AD and recovery of disease-related skin dysbiosis (54). Our data also mirrored previous research demonstrating nbUVB treatment is associated with increased *Corynebacterium* abundance (11). In addition to its potent anti-staphylococcal powers (55), this genus may influence CTCL skin activity depending on disease stage. As *Corynebacterium*-induced skin inflammation is contextually influenced by the host’s overall metabolic state and gut dysbiosis severity is tied to more advanced CTCL, further study of the *gut-skin* axis and *Corynebacterium* in CTCL is warranted (19, 56). Our findings clearly demonstrated a deep and unexplored network of ASVs arising from *Corynebacterium* genera, each with specific responses among the research interrogations performed longitudinally here. This strongly suggests that there is an as-yet unexplored diversity of responses to clinically-relevant interventions that manifest as changes at the sub-genus level.

Ours is the largest evaluation of the skin microbiota in CTCL to date and the first to evaluate the impact of a therapy on this microcosm within CTCL. The novel identification of genus-level shifts in the microbiome of non-lesional skin introduces the idea that the microbial profiles of these sites could be utilized to anticipate nbUVB responsiveness. While our study is limited by the heterogeneity and distribution of disease stage within our cohort, capturing the larger shifts in the microbiome across disease stage in CTCL has yet to be thoroughly documented. Just as transcriptional heterogeneity has been associated with CTCL (57), we expect the influence of the microbiome may vary on both inter- and intra-patient bases. Larger datasets may provide greater statistical power for conducting multivariate analyses. Furthermore, the likelihood that multiple bacterial species influence CTCL substantiate why shifts in some genera were identified in our cohort while others (e.g., *Bacillus*) were not found in our samples, despite their potential connection to CTCL tumorigenesis (24). Future analysis accounting for geographical variations in human microbiota composition is also warranted (58).

Microbes contribute to the impaired skin barrier integrity and altered local immune activity known of CTCL (59) and the extent of skin dysbiosis has been associated with disease stage (22). Here, we establish that the skin microbiome could also involve microbial biomarkers that predict nbUVB response, such as greater *Staphylococcus* relative abundance, yet we also recognize that patient heterogeneity will require further assessment in this research area. Larger and ideally multicenter analyses will validate these observations and expand upon their clinical translation.

## Data availability statement

The datasets presented in this study can be found in online repositories. The names of the repository/repositories and accession number(s) can be found below: https://www.ncbi.nlm.nih.gov/bioproject/PRJNA853302.

## Ethics statement

The studies involving human participants were reviewed and approved by the Northwestern University Institutional Review Board (STU00209226). The patients/participants provided their written informed consent to participate in this study.

## Author contributions

Conceptualization: XZ; Data Curation: XZ, TL, MH, and FV; Formal Analysis: GE, MB, DS, and SG; Funding Acquisition: XZ; Investigation: XZ, TL, MH, and FV; Methodology: SG, XZ, and PS; Project administration: XZ and JG; Resources: XZ, JG, SG, and MB; Software: MB and SG; Supervision: XZ, PS, and JG; Validation: XZ, MB, and SG; Visualization: MH and GE; Writing – original draft: MH and XZ; Writing – review and editing: MH, XZ, JG, MB, JC, SG, GE, FV, and TL. All authors contributed to the article and approved the submitted version.

## Funding

Supported by a Dermatology Foundation Medical Dermatology Career Development Award, Cutaneous Lymphoma Foundation Catalyst Research Grant, American Cancer Society Institutional Research Grant, and an institutional grant from the Northwestern University Clinical and Translational Sciences Institute (NUCATS) and the National Institutes of Health (NIH) (GRANT KL2TR001424).

## Acknowledgments

The authors would like to thank the patients who contributed to the study.

## Conflict of interest

The authors declare that the research was conducted in the absence of any commercial or financial relationships that could be construed as a potential conflict of interest.

## Publisher’s note

All claims expressed in this article are solely those of the authors and do not necessarily represent those of their affiliated organizations, or those of the publisher, the editors and the reviewers. Any product that may be evaluated in this article, or claim that may be made by its manufacturer, is not guaranteed or endorsed by the publisher.
