# Supplementary material for: Narrowband ultraviolet B response in cutaneous T-cell lymphoma is characterized by increased bacterial diversity and reduced Staphylococcus aureus and Staphylococcus lugdunensis
Source: Front Immunol. 2022 Nov 11;13:1022093. doi: 10.3389/fimmu.2022.1022093 (PMC9692126; doi:10.3389/fimmu.2022.1022093)
Supplement: Supplementary file 1 [file Table_1.docx]

***Supplementary Materials***

**1 Supplementary Table S1.** Detailed disease characteristics and treatment modalities of patients.

| **Sex** | **Age** | **Race** | **FST** | **CTCL Subtype** | **Stage** | **mSWATvisit 1** | **mSWAT visit 2** | **Other Treatments** |
| --- | --- | --- | --- | --- | --- | --- | --- | --- |
| ***nbUVB* *Responders*** | | | | | | | | |
| M | 49 | Asian | IV | MF | IB | 60 | 8 | TCS |
| M | 48 | White | II | MF | IB | 11 | 4 | TCS |
| M | 66 | White | II | Sézary syndrome | IV | 32 | 15 | None |
| M | 65 | White | II | MF | IA | 13 | 7 | TCS |
| F | 72 | Black | VI | MF | IIIA | 80 | 45 | TCS |
| F | 17 | White | III | FMF | IB | 26 | 7 | IFN-a |
| M | 55 | White | III | FMF | IB | 34 | 20.5 | TCS, tacrolimus |
| F | 71 | White | I | FMF | IB | 3 | 2 | TCS, bexarotene, IFNα-2β |
| M | 66 | White | III | MF | IIB | 11 | 4.5 | TCS, acitretin, imiquimod |
| M | 81 | White | III | MF | IB | 10 | 5 | Bexarotene |
| M | 72 | White | I | PCAETCL/  Gamma Delta | IIA | 38 | 18.5 | TCS |
| M | 54 | White | I | FMF/SMF | IB | 33 | 0 | TCS |
| M | 62 | White | I | MF | IB | 23 | 2 | None |
| F | 35 | Other | III | FMF | IB | 28 | 5 | None |
| ***nbUVB Non-Responders*** | | | | | | | | |
| F | 52 | White | II | PCAETCL | IB | 20 | 22 | Bexarotene, IFN-α |
| M | 71 | White | II | FMF | IIIB | 30 | 35 | TCS, acitretin |
| M | 55 | White | II | CD30+ LyP with systemic ALCL | IV | 80 | 159 | None |
| M | 57 | White | II | MF | IIA | 22 | 22 | None |
| F | 35 | White | III | MF | IB | 7.5 | 25 | TCS, bexarotene |
| M | 36 | White | III | FMF | IB | 3 | 4 | TCS |
| F | 73 | White | III | FMF | IIB | 15 | 18 | TCS, acitretin |
| M | 66 | Black | V | Sézary syndrome | IV | 10 | 20 | TCS |
| M | 73 | White | II | MF | IA | 12 | 22 | TCS, bexarotene |
| M | 46 | Black | V | MF | IIB | 60 | 65 | TCS |
| F | 63 | Black | V | FMF | IIB | 20 | 13 | TCS |
| ***Not treated with nbUVB*** | | | | | | | | |
| M | 68 | White | III | MF | IIB | 2.5 | 7 | Imiquimod |
| M | 37 | White | III | MF | IIB | 65 | 102 | None |
| M | 62 | White | II | PCAETCL | IIB | 21 | 25 | None |
| M | 65 | White | II | FMF | IIIA | 90 | 80 | TCS, prednisone |
| M | 67 | White | II | FMF/SMF | IB | 13 | 10 | TCS, IFN-α, acitretin |
| M | 68 | White | I | MF | IIIA | 10.5 | 14 | TCS |
| F | 76 | White | II | PCAETCL | IIB | 9 | 9 | TCS, urea 20%, acitretin |
| M | 65 | White | II | MF | IA | 5 | 4 | TCS |
| M | 59 | White | II | MF | IA | 3 | 6 | TCS, methotrexate |
| M | 83 | White | II | Sézary syndrome | IVA | 96 | 100 | TCS, methotrexate |
| M | 62 | White | II | MF | IIB | 3.5 | 6 | TCS |
| F | 66 | White | II | Sézary syndrome | IV | 21 | 28 | TCS, prednisone |
| F | 72 | Black | V | MF | IIIA | 85 | 85 | TCS |
| F | 46 | White | III | MF | IA | 14 | 15 | None |
| F | 58 | Asian | III | MF | IIB | 18 | 14 | TCS |

ALCL: anaplastic large cell lymphoma, CTCL: cutaneous T-cell lymphoma, F: female, FMF: folliculotropic mycosis fungoides, FST: Fitzpatrick skin phototype, IFN: interferon, LyP: lymphomatoid papulosis, M: male, MF: mycosis fungoides, mSWAT: Modified Severity- Weighted Assessment Tool, PCAETCL: primary cutaneous aggressive epidermotropic T-cell lymphoma, PUVA: psoralen and UVA light, SMF: syringotropic mycosis fungoides, TCS: topical corticosteroids
